# Multisystem inflammatory syndrome in children: a case series

**DOI:** 10.1590/1984-0462/2022/40/2021046

**Published:** 2022-04-04

**Authors:** Arianne Ditzel Gaspar, Gabriela de Sio Puetter Kuzma, Luana Amancio, Idilla Floriani, Vinicius Neves Bezerra, Gabriela Cristina Bortolon, Ana Paula Viana de Siqueira, Maura Peruchi Machado, Ana Cristina dos Santos Machado, Camila Faversani Camargo, Paulo Ramos David João

**Affiliations:** a Hospital Pequeno Príncipe, Curitiba, PR, Brazil.

**Keywords:** COVID-19, SARS-CoV-2 infection, SARS-CoV-2, Kawasaki Disease, Pediatrics, COVID-19, Infecção pelo SARS-CoV-2, SARS-CoV-2, Doença de Kawasaki, Pediatria

## Abstract

**Objective:**

To describe a case series of multisystem inflammatory syndrome in children (MIS-C) in a pediatric tertiary hospital.

**Methods:**

Patients under the age of 18 years who met MIS-C criteria of the Brazilian Ministry of Health (MH) and/or the Royal College of Paediatrics and Child Health (RCPCH) were included. A retrospective analysis was carried out by reviewing medical records and complementary exams.

**Results:**

Six pediatric patients with mean age of 126 months were admitted with fever associated with multisystem involvement: all of them had abdominal pain and diarrhea and two underwent appendectomy; 100% had coagulopathy and increased inflammatory markers; 83% had cardiovascular impairment and 60% required vasoactive drugs; 83% had mucocutaneous symptoms and 50% required ventilatory support by invasive mechanical ventilation or non-invasive ventilation. One patient showed coronary artery dilation on echocardiogram. All patients received empiric antibiotic therapies. SARS-CoV-2 IgG testing was positive in five patients. Treatment was performed after excluding infectious causes: five patients (83%) received intravenous immunoglobulin, five patients (83%) pulse methylprednisolone therapy and one (16%) Tocilizumab. One patient died. The average length of stay in Pediatric Intensive Care Unit (PICU) was seven days.

**Conclusions:**

These cases are added to the literature in construction of this emerging condition. Early diagnosis should be considered due to its potential severity.

## Resumo

**Objetivo:**

Descrever uma série de casos de síndrome inflamatória multissistêmica pediátrica (SIM-P) atendidos em um hospital terciário pediátrico.

**Métodos:**

Foram incluídos pacientes com idade menor que 18 anos que preenchessem os critérios de SIM-P do Ministério da Saúde (MS) e/ou do *Royal College of Paediatrics and Child Health* (RCPCH). Realizou-se análise retrospectiva por revisão de prontuário médico e de exames complementares.

**Resultados:**

Seis pacientes pediátricos com idade média de 126 meses, admitidos com quadro de febre associada a acometimento multissistêmico: todos apresentavam dor abdominal e diarreia e dois foram submetidos a apendicectomia; 100% apresentava coagulopatia e aumento das provas de atividade inflamatória; 83% apresentou comprometimento cardiovascular e 66% necessitou de drogas vasoativas; 83% apresentou sintomas mucocutâneos e 50% necessitou de suporte ventilatório por meio de ventilação mecânica invasiva ou não invasiva. Um paciente apresentou dilatação coronariana na ecocardiografia. Todos receberam antibioticoterapia empírica. Foi detectada sorologia de imunoglobina G positiva para SARS-CoV-2 em cinco pacientes. O tratamento foi realizado após a exclusão de causas infecciosas: imunoglobulina endovenosa em cinco pacientes (83%), pulsoterapia com metilprednisolona em cinco (83%) e Tocilizumabe em um (16%). Um paciente evoluiu para óbito. O tempo médio de internação em Unidade de Terapia Intensiva (UTI) foi de sete dias.

**Conclusões:**

Somam-se esses casos à literatura em construção para essa condição emergente. O diagnóstico deve ser considerado precocemente por seu potencial gravidade.

## INTRODUCTION

Multisystem inflammatory syndrome in children (MIS-C) emerged during the new coronavirus disease (COVID-19) pandemic, when children hospitalized in the United Kingdom (UK) and other countries from March to May 2020 developed fever and multisystem inflammation and, in severe cases, shock and multiple organ failure.^1^ The hypothesis of association with SARS-CoV-2 was then considered.^2^

MIS-C is known as an inflammatory condition that appears after the acute phase of infection, suggesting post-viral immune dysregulation that leads to a hyperinflammatory state.^3,4^ MIS-C shares characteristics with Kawasaki disease, toxic shock syndrome, bacterial sepsis, and macrophage activation syndrome. Adults with COVID-19- associated “cytokine storm” syndrome also had clinical and laboratory characteristics similar to familial hemophagocytic lymphohistiocytosis or macrophage activation syndromes, such as: elevations of serum ferritin levels, liver enzymes, soluble interleukin-2 (IL-2) (sCD25) receptor, D-dimer, clotting times (prothrombin time and activated thromboplastin time) and lactic dehydrogenase (LDH), thrombocytopenia and lymphopenia.^5^

So far there are no universally accepted protocols for MIS-C management, so many organizations have published their own protocols.^3–7^ In Brazil, the diagnosis of MIS-C is established by the criteria proposed by the Ministry of Health (MH): hospitalized cases with presence of high (>38° C) and persistent (≥3 days) fever in children and adolescents (up to 19 years old) and at least two of the following signs and/or symptoms: non-purulent conjunctivitis or bilateral skin lesion or signs of mucocutaneous inflammation (oral, hands or feet); hypotension or shock; manifestations of myocardial dysfunction, pericarditis, valvulitis, or coronary abnormalities – including echocardiogram findings, elevated troponin or N-terminal B-type natriuretic peptide (NT-proBNP) – evidence of coagulopathy (due to increased prothrombin time, partially activated thromboplastin time, or D-dimer); acute gastrointestinal manifestations (diarrhea, vomiting or abdominal pain). Patients must have high inflammation markers [erythrocyte sedimentation rate (ESR), C-reactive protein (CRP) or procalcitonin (PCT), among others), any other causes of infectious and inflammatory origin, including bacterial sepsis, staphylococcal or streptococcal shock syndromes must be ruled out. Also, there must be evidence of COVID-19 (molecular biology, positive antigenic or serological test) or history of contact with a COVID-19 case.^5,6^

The Royal College of Pediatrics and Child Health (RCPCH) case definition is described below. 1) A child presenting with persistent fever, inflammation (neutrophilia, elevated CRP and lymphopenia) and evidence of single or multi-organ dysfunction (shock, cardiac, respiratory, renal, gastrointestinal or neurological dysfunction) with additional features: clinical (all—persistent fever >38.5°C; most—oxygen requirement, hypotension; some—abdominal pain, confusion, conjunctivitis, cough, diarrhea, headache, lymphadenopathy, mucus membrane changes, neck swelling, rash, respiratory symptoms, sore throat, swollen hands and feet, syncope, vomiting); laboratory tests (all—abnormal fibrinogen, absence of potential organisms other than SARS-CoV-2, high CRP, high D-dimers, high ferritin, hypoalbuminemia, lymphopenia, neutrophilia (in most, neutrophils in some); some—acute kidney injury, anemia, coagulopathy, high IL-10 or IL-6 (if available), neutrophilia, proteinuria, raised creatine kinase (CK), raised LDH, raised triglycerides, raised troponin, thrombocytopenia, transaminitis); echocardiogram/electrocardiogram (ECG)—myocarditis, valvulitis, pericardial effusion, coronary artery dilatation; Chest X-ray: patchy symmetrical infiltrates, pleural effusion; abdominal ultrasound: colitis, ileitis, lymphadenopathy, ascites, hepatosplenomegaly; chest computed tomography scan (CT): may demonstrate coronary artery abnormalities. This may include children meeting full or partial criteria for Kawasaki disease. 2) Exclusion of any other microbial cause, including bacterial sepsis, staphylococcal or streptococcal shock syndromes, infections associated with myocarditis such as enterovirus (waiting for results of these investigations should not delay seeking expert evaluation). 3) Reverse transcriptase followed by polymerase chain reaction (RT-PCR) for SARS-CoV-2 may be positive or negative.^7^

Patients can rapidly progress to severe forms of the disease with acute respiratory failure, acute kidney disease, hypotension, acute heart failure and shock. Thus, the goal of treatment is to reduce the hyper inflammatory state and restore organs and systems functions, reducing sequelae such as coronary artery lesions and cardiac dysfunction, in addition to reducing mortality.^5^

As this is a new and potentially serious condition coupled with the need for more knowledge on the subject, we present a series of six cases of MIS-C treated at a tertiary pediatric hospital in southern Brazil.

## METHOD

A case series descriptive study was carried out at Hospital Pequeno Príncipe. The hospital is located in southern of Brazil and has more than 370 hospital beds and 68 intensive care beds. Patients under 18 years old who met the MH and/or RCPCH MIS-C criteria were included.^5,7^ The study was approved by the Ethics Committee for Research Involving Human Beings of the Hospital Pequeno Príncipe (number 4,512,932).

## CASES DESCRIPTION

### Case 1

A 12-year-old male brown patient with a past history of controlled asthma presented with a 3-day history of abdominal pain, persistent fever, vomiting and diarrhea. He was submitted to appendectomy after the initial diagnosis of acute appendicitis. After three days, he was transferred to the PICU with severe abdominal pain, high fever, diffuse myalgia and altered mental status. He also had, skin rash and bilateral pleural effusion at PICU admission. Notable laboratory findings included high D-dimer, high IL-6, raised acute phase reactants, elevated troponin, and hypoalbuminemia. The electrocardiogram showed increased QTc (560 ms). Echocardiogram was normal. There was no need for inotropic support. The appendix pathology reported lymphoid hyperplasia. SARS-CoV-2: immunoglobulin G (IgG) testing was positive and immunoglobulin M (IgM) was negative. SARS-CoV-2 RT-PCR testing was negative. He received acetylsalicylic acid (ASA) and intravenous immunoglobulin (IVIG) and presented a good clinical evolution.

### Case 2

A 13-year-old Caucasian female patient, with no chronic medical conditions, was admitted with a 4-day history of fever and abdominal pain. She was submitted to appendectomy due to hypothesis of acute appendicitis diagnosis. The appendix pathology report showed diffuse lymphoid hyperplasia and serous vascular congestion. SARS-CoV-2 RT-PCR testing was positive one month earlier. On the first postoperative day, she evolved with severe abdominal pain, vomiting, diarrhea, tachypnea, and hypotension, and was referred to the PICU. She presented skin rash, conjunctival hyperemia, and neck swelling. Notable laboratory findings on admission included high D-dimer and elevated inflammatory markers, thrombocytopenia, coagulopathy, elevated urea and creatinine, and hypoalbuminemia. SARS-CoV-2 IgG testing was positive and IgM testing was negative. The patient evolved with respiratory failure and need of invasive mechanical ventilation (IMV) and vasoactive drugs. Lumbar puncture was not performed due to clinical instability. Chest CT scan showed bilateral pleural effusion and bilateral pulmonary opacities with pulmonary edema pattern. Her echocardiogram demonstrated dilated coronary arteries and IVIG infusion was started. On the following day, the patient evolved to significant clinical worsening, requiring high-dose vasoactive drugs; pulse methylprednisolone therapy was started. Although renal replacement therapy was indicated, it has not been started due to severe hypotension. In the context of metabolic acidosis, renal failure, and refractory shock—receiving epinephrine, norepinephrine, and high-dose vasopressin—, she evolved with progressive increase in heart rate and QRS duration and, after a few hours, with left bundle branch block and accelerated idioventricular rhythm. The patient presented a cardiorespiratory arrest unresponsive to cardiopulmonary resuscitation and progressed to death.

### Case 3

A 14-year-old Caucasian male patient, with no chronic medical conditions, was admitted with a 5-day history of fever, abdominal pain, vomiting, diarrhea and skin rash. SARS-CoV-2 RT-PCR testing was positive four days prior to admission, and he had no respiratory symptoms. Empirical antibiotic therapy, volume expansion and epinephrine infusion were started due to refractory hypotension. Notable laboratory findings were lymphopenia (507/uL), high D-dimer, elevated inflammatory markers, and ferritin, besides an increased international normalized ratio (INR), acute renal injury, and coagulopathy, and elevated urea and creatinine. Abdominal ultrasound was normal. The echocardiogram, performed during the infusion of vasoactive drugs, was normal. Troponin was elevated and ECG showed prolonged QTc. Patient received ASA and then prophylactic enoxaparin. IVIG infusion was started. Clinically the patient remained with signs of shock, so milrinone infusion and pulse methylprednisolone therapy were started. After the first dose of pulse therapy, patient presented a significant clinical improvement. Maintenance corticoid therapy was initiated. The patient evolved with progressive clinical and laboratory improvement.

### Case 4

A 10-year-old Caucasian female patient, with no chronic medical conditions, was admitted with a 5-day history of fever and abdominal pain associated with a 3-day history of skin rash and altered mental status. Notable laboratory findings on admission included thrombocytopenia, anemia, lymphopenia (789/uL), hypoalbuminemia, mixed acidosis, acute kidney injury, elevated inflammatory markers, D-dimer and troponin. elevated CRP, urea, and creatinine. Empirical antibiotic therapy was started and the patient was transferred to the PICU due to volume-refractory shock. Ferritin, ESR, D-dimer and troponin levels were high. Due to history of abdominal pain, a CT scan of the abdomen was performed, showing moderate bilateral pleural effusion and ascites, in addition to bilateral renal low uptake with multiple small hypodense parenchymal images (maximum diameter of 26 mm), suggestive of inflammatory or infectious etiology. Pediatric surgeons and interventional radiologists concluded that the lesions could not be reached by biopsy and, in that clinical context, were suggestive of an inflammatory etiology. Both blood and urine cultures were negative, as well as antigen and serology testings for dengue and leptospirosis serology testing. Patient required non-invasive mechanical ventilation (NIV). Chest CT scan showed moderate bilateral pleural effusion, interstitial pulmonary edema, and basal atelectasis. Echocardiogram showed moderate left ventricular dysfunction, and ECG showed long QTc. It was started norepinephrine and dobutamine. SARS-CoV-2 IgG testing was positive, and IgM and RT-PCR testing were negative. She received pulse methylprednisolone therapy and IVIG infusion, and presented clinical and laboratory improvement.

### Case 5

A 6-year-old Caucasian male patient, previously healthy, was admitted with a 5-day history of high fever of up to 40° C associated with headache, vomiting, bilateral conjunctival hyperemia and abdominal pain for one day. He had history of contact with his grandmother infected with SARS-CoV-2 a month before. The next day after admission, he was transferred to the PICU for altered mental status and need of oxygen therapy. Laboratory tests were notable for elevated D-dimer, CRP and ESR, increased INR, metabolic acidosis and lymphopenia (142 U/L). SARS-CoV-2 IgG testing was positive and IgM testing was negative. Patient received one dose of pulse methylprednisolone therapy and had significant improvement in his general condition. On the following day, he evolved with hypotension associated with mild systodiastolic dysfunction and vasoactive drugs and IVIG infusion were started. Pulse methylprednisolone therapy was maintained for five days. Despite a normal initial troponin value, the patient had a mild elevation during hospitalization: 50 pg/mL [reference value (RV)5 – 42pg/mL). He required IMV for one day and NIV for one day. After the end of pulse therapy, patient continued to have respiratory distress and drowsiness, besides requiring vasoactive support; Tocilizumab (12mg/kg) was then indicated. Serum IL-6 dosage was high: 6.5 pg/ml (RV<5.9). After that, he evolved with progressive clinical and laboratory improvement and normalization of the echocardiogram.

### Case 6

A 6-year-old Caucasian male patient was admitted to the ward with a 2-day history of abdominal pain associated with fever, nausea and vomiting. Laboratory tests showed blood count with left shift, high D-dimer and CRP, and procalcitonin level suggestive of systemic inflammation. Abdominal ultrasound showed dilated loops and mesenteric lymphadenitis. SARS-CoV-2 serology testing was negative. After discarding infectious causes, pulse methylprednisolone therapy was started with good clinical response and improvement in the evidence of inflammatory activity.

All cases reported were in the acute MIS-C phase, as described. These patients remain under outpatient follow-up at our service. Table 1 shows clinical and therapeutic data, as well as length of stay in the PICU. Table 2 shows the results of imaging tests of the study patients, and Tables 3 and 4 show the results of the initial laboratory tests.

**Table 1.** Clinical data and treatment administered to patients diagnosed with pediatric multisystem inflammatory syndrome in a pediatric hospital.

Data	Case 1	Case 2	Case 3	Case 4	Case 5	Case 6
Age (months)	150	169	170	126	80	65
Sex	M	F	M	F	M	M
Abdominal pain	+	+	+	+	+	+
Conjunctival hyperemia	+	+	+	+	+	–
Rash	+	+	+	+	–	–
Diarrhea	+	+	+	+	+	+
Vomit	–	+	+	+	+	+
Hypotension	+	+	+	+	+	–
Ventilatory support	AA	IMV	NC	NC; NRM; NIV	IMV; NIV	AA
Antimicrobials	Gentamicin Metronidazole Ceftriaxone Piperacillin-tazobactam	Gentamicin Metronidazole Ceftriaxone Clindamycin	Metronidazole Ceftriaxone Clindamycin	Ceftriaxone Clindamycin	Metronidazole Ceftriaxone Clindamycin	Ceftriaxone
Vasoactive drugs	*	Adrenaline Noradrenaline Vasopressin	Adrenaline Milrinone	Noradrenaline Dobutamine	Dobutamine Noradrenaline Adrenaline Milrinone	*
Pulse therapy**	*	1 day	1 day	1 day	5 days	1 day
IVIG	2 g/kg	2g/kg	2g/kg	2g/kg	2g/kg	*
Tocilizumab	*	*	*	*	12mg/kg	*
Length of stay at PICU	11 days	2 days	7 days	7 days	8 days	*

+: alteration present; -: no alteration; M: male; F: female; AA: ambient air; IMV: invasive mechanical ventilation; NC: nasal cannula; NIV: non-invasive ventilation; NRM: non-rebreathing mask; PICU: Pediatric Intensive Care Unit; *: not done; **: pulse therapy: methylprednisolone 30 mg/kg/dose or 1g; IVIG: intravenous immunoglobulin.

**Table 2.** Additional tests in patients diagnosed with pediatric multisystem inflammatory syndrome in a pediatric hospital.

Data	Case 1	Case 2	Case 3	Case 4	Case 5	Case 6
Chest X-ray	Normal	Bilateral alveolar opacities	Normal	Bilateral alveolar opacities	Normal	Normal
Chest ultrasound	*	Laminar PE; bibasal atelectasis.	Laminar PE; Basal consolidation/atelectasis.	Bilateral PE. Bilateral sparse atelectasis.	*	*
Chest CT	*	Enlarged cardiac volume, enlarged pulmonary trunk and main pulmonary arteries caliber. Smooth thickening of interlobular septa. Peribronchovascular interstitium thickening. Ground-glass opacities; atelectasis; diffuse pulmonary opacities suggestive of pulmonary edema.	Small bilateral PE. Basal atelectasis. Ground-glass opacities predominantly subpleural in the lung bases.	Moderate bilateral PE. Interstitial pulmonary edema. Basal atelectasis.	Bilateral PD. Diffuse ground-glass opacities. Bilateral and centrilobular opacities in the left upper lobe.	*
Echocardiogram	Normal. LV EF 70%.	LV EF 68%; coronary arteries origin dilation**.	Normal. LV EF 70%**.	Moderate LV dysfunction; CI: 2,6L/min/m^2^ LV EF 54%.	Mild systolic-diastolic dysfunction; LV EF 57% SF 29%.	*
Abdominal ultrasound	Small PE	Increased echogenicity of adipose planes and liquid collected at RIF.	Normal	Small/moderate amount of free fluid in the pelvis.	Usual-looking lymph nodes in RIF. Cecal appendix not visualized.	Dilated loops. Mesenteric adenitis.

LV: left ventricle; EF: ejection fraction; SF: shortening fraction; CT: computed tomography; PE: pleural effusion; RIF: right iliac fossa; LV: left ventricle; CI: cardiac index; *: not performed; **: echocardiogram performed while using vasoactive drugs.

**Table 3.** Notable initial laboratory findings of patients admitted with pediatric multisystem inflammatory syndrome in a tertiary pediatric hospital.

	D-dimer (ng/L) RV <500	Ferritin (ng/mL) RV 7-140	Fibrinogen (mg/dL) RV 200-393	Troponin (pg/mL)	Procalcitonin (ng/mL) RV<0,5	Albumin (g/dL)	Lactate (mmol/L)	Creatinine (mg/dL)	Urea (mg/dL)	ESR (mm/1h)	CRP (mg/L) RV<10
Case 1	2,570	154	314	30.2 (RV <19.8)	*	2.1	0.9	0.4	22	55	246
Case 2	2,540	355	314	*	*	2.7	2.3	0.5	26	88	239
Case 3	1,738	1,676	408	113.5 (RV <19.8)	*	2.8	6.8	1.4	85	40	403
Case 4	1,361	351.4	574	427.7 (RV<11.6)	1.8	1.9	0.8	2.2	119	33	335
Case 5	984.24	149.1	671	5 (RV 5-42)	*	2.6	1.3	0.3	34	41	223.5
Case 6	798	*	*	<2.3 (RV<19.8)	2.4	*	1.6	0.3	19	*	209

ESR: erythrocyte sedimentation rate; CRP: C-reactive protein; RV: reference value; *: not collected.

**Table 4.** Notable initial laboratory findings of patients admitted with pediatric multisystem inflammatory syndrome in a tertiary pediatric hospital.

	Platelet count (/μL)	ISR	Ht (%)	Hb (g/dL)	Leukocytes (/uL)	Triglycerides (mg/dL)	GOT (U/L)	GPT (U/L)	SARS-CoV-2 IgM and IgG Serology Tests	SARS-CoV-2 RT-PCR Test
Case 1	143,000	1.8	35	11	7,310	225	30	20	IgG +; IgM -	Negative
Case 2	80,000	1.5	35	12	10,920	107	111	137	IgG +; IgM -	External: negative
Case 3	222,000	1.4	36.9	13	10,140	*	32	26	IgG +; IgM -	External: positive Hospitalized: negative
Case 4	80,000	1.5	26.6	8.9	7,890	299	14	17	IgG +; IgM -	Negative
Case 5	229,000	1.4	35	11.8	5,350	*	37	20	IgG +; IgM -	External: negative
Case 6	236,000	1.2	36	12	4,570	*	34	17	IgG -; IgM -	*

INR = international normalized ratio; Ht: hematocrit; Hb: hemoglobin; GOT: glutamic-oxaloacetic transaminase; GPT: glutamic-pyruvic transaminase; *: not collected.

## DISCUSSION

MIS-C is a new condition that emerged along with the COVID-19 pandemic. Its diagnosis should be considered in every child or adolescent who presents persistent fever, increased inflammatory markers and evidence of single or multi-organ dysfunction after excluding infectious causes that could justify the condition.^1^

In a Brazilian multicenter study that evaluated a cohort of patients with MIS-C, 55% had SARS-CoV-2 infection detected by RT-PCR, serological testing, or both, and almost half of patients had a history of contact with a COVID-19 case.^8^ One patient of the present sample had no laboratory evidence of prior SARS-CoV-2 infection. The definition of MIS-C by the RCPCH does not entail the mandatory identification of SARS-CoV-2 for the diagnosis of this condition.^7^

In the present series, all patients had prominent gastrointestinal symptoms. Most of them also had mucocutaneous alterations (83% conjunctival hyperemia and 66% skin rash). These findings are described in the literature: in a systematic review carried out in 2020 with patients with MIS-C, 87% had gastrointestinal symptoms, 73% had dermatological/mucocutaneous symptoms and 71% had cardiovascular symptoms.^2^ Similarly, in an evaluation of 17 patients in New York, 14 had gastrointestinal symptoms, being common mucocutaneous symptoms, in addition to 13 cases of shock on admission.^9^

In the context of high prevalence of gastrointestinal symptoms in these individuals, we noted that two of our patients underwent appendectomy without findings consistent with acute appendicitis on pathology report. This seems to occur with some frequency: in a multicenter study of 25 patients in France, two children underwent emergency laparotomy for suspected appendicitis.^10^

In a systematic review of MIS-C, all studies reported elevated levels of CRP and other inflammatory markers in at least 75% of patients in each study, suggesting that hyperinflammatory state is a primary feature of MIS-C.^2^ As shown in Table 3, all patients in the present series had evidence of increased inflammatory activity.

It is known that ventricular dysfunction and cardiogenic shock can occur in more than 50% of patients and markers of cardiac dysfunction are increased in more than 75% of cases.^5,8,11^ In an observational study of 15 children with MIS-C in the UK, all patients showed increased inflammatory/cardiac markers (CRP, ferritin, troponin, creatine phosphokinase, pro-BNP): in 10 patients, transient valve regurgitation was observed, ejection fraction was reduced in 80% and shortening fraction was reduced in 53%, with complete resolution in almost all patients except two.^12^ Still, it is important that these patients have medium and long term follow-up as the sequelae after the acute phase remains uncertain.^3,5,11^ In the present study, 83.3% of the patients presented cardiac alteration – manifested by myocardial dysfunction, electrocardiographic alteration, hypotension, troponin increase or coronary artery abnormality – which reinforces the relevance of cardiac involvement in MIS-C cases. It is noteworthy that 50% of patients described here had increased Qtc.

Considering that many patients come to medical care with clinical symptoms that mimic sepsis, while there is no diagnosis confirmation, empiric antibiotic therapy should be started, what has been done in all patients in the present study. Moreover, in most cases the use of inotropic drugs is necessary, and these patients should generally be admitted to intensive care units, which occurred with five of the six patients in the present case series.^1^

In the present study, all patients admitted to the PICU received IVIG infusion, and its immediate administration is recommended by many institutions: it should be considered in cases with moderate and severe presentations and in cases meeting complete or partial criteria for Kawasaki Disease and/or macrophage activation.^7,11^ Furthermore, it should also be considered in cases that mimic toxic shock syndrome, which proved to be refractory to conventional treatment, and may be repeated in cases refractory to the first dose.^5,11^

Regarding the use of corticosteroids: their use in cases of MIS-C has been described in many studies, as an attempt to reduce the exacerbated inflammatory response.^8^ Therefore, they should be considered along with IVIG in severe cases and in those that were refractory to its infusion. Corticosteroids can be administered as pulse therapy (10-30 mg/kg/day of methylprednisolone for one to three consecutive days), followed by 2 mg/kg/day for five days, and the dose should be gradually decreased over two to three weeks.^5^ Their use should be considered in the presence of myocardial involvement, even if minimal, and also in mild cases, such as Case 6.1 In the present study, only one patient has not receive corticosteroid therapy; among those who received it, four did so for one day (one of them died early, which limited the assessment) and one for five days. Since some of the cases reported here occurred in the circumstance of the initial MIS-C reports in the medical community, knowledge about the ideal therapeutic approach was scarce, which justifies the heterogeneity in patients’ therapeutic management.

Recently, a retrospective cohort study evaluated the use of isolated IVIG versus IVIG in association with methylprednisolone in the treatment of MIS-C. The use of immunoglobulin associated with corticosteroids reduced the risk of treatment failure [Odds Ratio (OR) 0.25], the need for second-line therapy (OR 0.19), hemodynamic support (OR 0.21), left ventricular dysfunction after initial therapy (OR 0.20) and the length of PICU stay. However, there was no standardization in the prescription of corticosteroids among patients, so further studies are needed to determine the optimal dose and duration of treatment with corticosteroids.^13^

As in Case 5, in which the use of an anti-IL-6 agent was indicated, immunomodulation with biological agents has been used and seems to play an important role in adult patients with severe COVID-19, to whom the use of Tocilizumab was described for pharmacological inhibition of this interleukin.^8,11^

Despite being a new condition, the diagnosis of MIS-C should be considered early by professionals due to its potential severity. The existence of risk factors for its development remains uncertain. Diagnostic suspicion and clinical management must be performed early in order to avoid negative outcomes. Follow-up should be continued after hospital discharge. There is need of further studies comparing therapeutic options and specific indications in MIS-C management.
